# Do codes of ethics and position statements help guide ethical decision making in Australian immigration detention centres?

**DOI:** 10.1186/s12910-019-0392-8

**Published:** 2019-07-23

**Authors:** Ryan Essex

**Affiliations:** 1 The University of Greenwich, London, UK; 2 Barts Health NHS Trust, London, UK; 3 The University of Sydney, Sydney, Australia

**Keywords:** Refugee, Asylum seeker, Immigration detention, Codes of ethics, Healthcare, Clinical ethics, Human rights, Migration

## Abstract

Australian immigration detention has been called state sanctioned abuse and a crime against humanity. The Australian healthcare community has been closely involved with these policies, calling for their reform and working within detention centres to provide healthcare. As well as having a devastating impact on health, immigration detention changes the scope and nature of healthcare, with its delivery described as a Sisyphean task. In this article I will explore the guidance that is available to clinicians who work within detention centres and argue that codes, guidelines and positions statements provide little help in relation to ethical decision making. First I will outline guidance that can be found in codes of ethics and position statements, focusing on particularly relevant principles, such as advocacy, clinical independence and the clinicians’ relationship to human rights. I will then highlight the disparity between this guidance and the delivery of healthcare within detention by drawing on the testimony of clinicians who formerly worked in these environments. While this disparity should be cause for alarm and at a minimum call into question how codes and positions statements are being used (if at all), there are more fundamental reasons why codes and position statements fail to provide guidance in these circumstances. I will outline a more general criticism of codes of ethics and use this to suggest a way forward, including looking beyond codes and position statements to guide action within Australian immigration detention.

## Background

### Australian immigration detention

Immigration detention has been one of the most contentious contemporary political issues in Australia for over two decades. Onshore detention was introduced in 1992, while offshore detention on Manus Island (Papua New Guinea) and Nauru were re-introduced in 2012. Those who arrived by boat after 2013 were sent offshore and given no opportunity to resettle in Australia [1]. In October 2015, the Nauru government announced that they would be processing all remaining asylum seekers who would no longer be confined within the detention centre. This was announced only days before an Australian High Court challenge, with the opening of the centre forming a key part of the government’s defence [2, 3]. In April 2016, Papua New Guinea’s Supreme Court ruled that detention on Manus Island was illegal [4]. The centre was formally closed in late 2017. While centres on Manus Island and Nauru are now both “opened” and despite a resettlement deal being struck with the United States, to this day, hundreds of people remain on Manus Island and Nauru with little or no news on resettlement or safety [5]. Amid ongoing protest and increasing political pressure, in early 2019 the government announced it would remove children from Nauru [6]. While the government claimed that all children were released from onshore detention in 2016, these claims have been proven to be false and misleading [7].

Numerically, these policies have resulted in the detention of tens of thousands of men, women and children both onshore and offshore. While numbers have declined recently, the number of people detained offshore peaked in April 2014, when 2,450 people (including 190 children) were detained on both Manus Island and Nauru [8]. Prior to the introduction of offshore processing there were 9,256 people in onshore immigration detention, including 1,820 children in June 2013 [9].

Conditions within detention centres (and on Manus Island and Nauru, since centres were opened) have been unsafe and violent. Multiple inquiries have provided details on widespread physical and sexual abuse, violence, riots, self-harm and suicidal behaviour [10]. As can be imagined with these conditions Australian immigration detention violates or impinges upon almost all human rights and international legal instruments to which Australia is signatory [11] including the right “to be free from torture or cruel, inhuman or degrading treatment.” [12]. The impact of these policies on health and wellbeing has also been well established, with all studies, testimony and evidence from inquiries suggesting that detention has a devastating impact on the health of detainees [13–15].

Rather than reform these policies or engage constructively with critics the Australian government has attempted to shut down debate and increase secrecy surrounding these policies. Journalists and contractors have been raided [16, 17], legislation was passed (but subsequently amended) that criminalised disclosures from staff [18] and the government has been belligerent toward human rights organisations [19, 20]. The government continues to justify this approach as a means to deter further asylum seeker boat arrivals [21, 22]. The damage these policies do and their deliberate nature have led many to call them crimes against humanity [23] and liken them to torture [24].

### Healthcare in Australian immigration detention

Healthcare has been provided within Australian immigration detention for over two decades. While the Australian government has maintained that healthcare is provided to a standard equivalent to that found in the broader Australian community, this is simply untrue [25]. Not only is this system antithetical to health, wellbeing and healthcare, these environments drastically alter the nature and scope of healthcare [25–27]. The delivery of healthcare has been described as a Sisyphean task [28]. In addition to the testimony that is presented below, there are a number of examples that speak to this point. The government has interfered in diagnoses [29], sought medical information for political purposes [30] and deported senior staff from offshore locations [31]. The government has also rejected cooperative efforts to improve healthcare [32]. There has also been multiple accusations about misconduct made against healthcare contractors [33]. For those offshore who need medical assistance, the government has sought to limit medical transfers to the mainland, which has had fatal consequences [34]. Like its approach more generally, the Australian government has been belligerent in the face of criticism and the relationship with the healthcare community could best be described as antagonistic, with the government openly dismissive and hostile to medical advice and calls for reform.

While the healthcare community has debated a boycott [35] and engaged in broader efforts to bring about systemic change, clinicians continue to work in these environments and are likely to do so into the foreseeable future.

The roles of clinicians working within detention have been discussed and debated for over two decades. Many have written about their experiences in the system [36, 37], some have testified at inquiries [14], while others have gone to the media [38, 39]. The bioethics literature has long discussed how clinicians should engage with this system [40–42] as have professional healthcare bodies [43]. While there is no consensus, there has been little critical reflection on existing literature and in particular the guidance provided by professional health care bodies in codes, guidelines and position statements. Sanggaran and Zion [44] have noted that current codes and position statements only serve to highlight “the chasm between acceptable standards of medical care and what we know is being practised in immigration detention”.

Below I expand on this observation and argue that codes, guidelines and positions statements authored by professional healthcare bodies provide little guidance in relation to clinical and ethical decision making. First I will outline guidance that can be found in codes of ethics and position statements, focusing on particularly relevant principles, such as clinical independence and the clinicians’ relationship to human rights. I will then highlight the disparity between this guidance and the delivery of healthcare within detention by drawing on the testimony of clinicians who formerly worked in these environments. While this disparity should be cause for alarm and at a minimum call into question how codes and positions statements are being used (if at all), there are more fundamental reasons why codes and position statements have limited utility in these circumstances. I will outline a more general criticism of codes of ethics and use this to suggest a way forward, including looking beyond codes and position statements to guide action within Australian immigration detention.

## Main text

### Codes of ethics

Below I will discuss four codes of ethics: The Australian Medical Association’s (AMA) Code of Ethics [45], the Australian Psychological Societies’ (APS) Code of Ethics [46], the International Council of Nurse’s Code of Ethics [47] and the Royal Australian and New Zealand College of Psychiatrist’s (RANZCP) Code of Ethics [48]. These codes represent the overwhelming majority of clinicians who have worked within the system and have been produced by professional bodies who have been active in discussions related to health and healthcare within Australian immigration detention.

While each code differs in scope and content, all set out to guide action. Some are focused on day to day clinical activity while others contain broad, aspirational principles. All discuss a number of fundamental ethical principles such as autonomy, informed consent and confidentiality. It is beyond the scope of this article to discuss each code in detail. The below discussion will focus on principles and statements that are particularly relevant for those working within detention and which have been identified as particularly problematic in the literature. This includes statements related to advocacy, clinical independence, managing multiple and conflicting relationships and human rights.

The AMA Code of Ethics [45] contains a range of ethical rules and principles. It discusses doctors’ relationship with their patient, the profession and society. Importantly it addresses the issue of clinical independence, calling for doctors to “[u] phold professional autonomy and clinical independence and advocate for the freedom to exercise professional judgement in the care and treatment of patients without undue influence by individuals, governments or third parties”. Despite having a section entitled human rights, this code does not make a general statement calling for doctors to uphold and protect human rights. It does however call for patients to be treated with dignity and calls on doctors not to, “countenance, condone or participate in the practice of torture or other forms of cruel, inhuman or degrading procedures”.

The APS Code of Ethics [46] is based on three broad ethical principles. Two are of particular relevance for practice in immigration detention. First, respect for the rights of dignity of people and peoples. This principle calls on psychologists to uphold autonomy, maintain confidentiality and seek informed consent. Second, integrity outlines standards related to the character of psychologists, calling for them to “exercise their power appropriately and honour this position of trust”. This principle also addresses the conduct expected in response to conflicts of interest and other ‘multiple relationships’. It calls for psychologists to “refrain from engaging in multiple relationships that may: a) impair their competence, effectiveness, objectivity, or ability to render a psychological service; (b) harm clients or other parties to a psychological service”. This code explicitly calls for psychologists to promote equity and protect peoples “human rights, legal rights, and moral rights”.

In 2018, the Nursing and Midwifery Board of Australia adopted the International Council of Nurses Code of Ethics (2012). This code outlines how nurses should approach their relationship and roles with patients, society, the profession and co-workers. It is the shortest and arguably the least prescriptive code reviewed here. It too discusses conduct related to confidentiality, autonomy and patient choice, informed consent and competency. This code also makes a number of statements in relation to nursing’s relationship with human rights and social justice, stating that “[t] he nurse shares with society the responsibility for initiating and supporting action to meet the health and social needs of the public, in particular those of vulnerable populations” and that “[i] nherent in nursing is a respect for human rights, including cultural rights, the right to life and choice, to dignity and to be treated with respect.”

The RANZCP Code of Ethics (2018) contains eleven principles, including guidance related to psychiatrists conduct in relation to patient autonomy, privacy and confidentiality, informed consent, the use of their professional skills and knowledge. While the code does not discuss human rights explicitly it calls on psychiatrists to “not participate in the practice of torture or cruel, inhuman or degrading interrogation, treatment or punishment”. It also calls for caution when negotiating multiple relationships, stating, “[p]sychiatrists’ primary responsibility is to patients. Particular care is needed when this conflicts with responsibility to an employer or government. If clinical services fall below acceptable standards, psychiatrists have a duty to advocate for services and take appropriate action”. It goes on to say that in exceptional circumstances psychiatrists may have to “dissociate themselves from such services”. This code also provides scope for advocacy and in one further point particularly relevant to healthcare in Australian immigration detention, it states that, “[p] sychiatrists should provide an adequate standard of care regardless of the legal status of patients or the setting in which they are being treated”. The RANZCP Code of Ethics [48] is supplemented by the RANZCP Professional Practice Guideline 12: Guidance for Psychiatrists Working in Australian Immigration Detention Centres [49]. This guidance focuses on “key ethical and professional practice issues that psychiatrists may encounter when working with asylum seekers in all forms of immigration detention”. Like the code of ethics, this guidance again calls for psychiatrists to refuse to participate in cruel, inhuman or degrading treatment and maintain patient confidentiality. It re-enforces the idea that a psychiatrists primary responsibility is to their patients, stating, “[p] sychiatrists should always act in the best interests of their patients, with respect for the essential humanity and dignity of every patient”. In regards to clinical independence, this guidance states that “[p] sychiatrists should have the opportunity to practice their speciality at the highest level of excellence”. It also discusses advocacy, calling for psychiatrists to advocate for their patients, which it says include “advocating for the patient to be managed in a less restrictive setting, to be transferred to another environment including inpatient psychiatric treatment or to have their immigration determination expedited”. Importantly this guidance also calls on psychiatrists to “advocate for broader structural or systemic” change.

### Position statements

Supplementing the above codes of ethics are a number of position statements. Below I will discuss the AMA [50], Royal Australian College of Physicians (RACP) [51] and APS [52] positon statements on the health and healthcare of refugees and asylum seekers. Each sets out to do at least one of two things. The first is to make explicit the position of the professional body on issues as they relate to refugee and asylum seekers in Australia and the second is to provide clinical and ethical guidance as to the standard of care that should be provided. Like the codes of ethics discussed above, all vary in scope and content but have a number of common themes. All acknowledge the harm created and perpetuated by Australian immigration detention and call for significant reform, making demands of the Australian government. The RACP [51] call for the abolition of immigration detention. The APS [52] call for an end to the detention of children, while the AMA [50] call for the use of detention as a last resort only, and only for limited periods of time.

The only professional body to explicitly question the utility of providing guidance and defining a standard of care has been the RACP [51]. They state that while they support clinicians in their roles, they also acknowledge “the significant ethical issues related to providing care in detention, and the tension in defining a standard of care”. They explicitly state that, “[t] his statement does not provide recommendations regarding detention health facilities, as the evidence shows held detention has a significant and detrimental impact on health and wellbeing, and the RACP does not condone held detention”. Below, I will focus on the statements that are intended to guide clinical action and that are particularly relevant for healthcare within Australian immigration detention.

The AMA [50] and APS [52] attempt to provide clinical and ethical guidance for clinicians and attempt to outline a standard of care which should be provided. This includes a mix of ethical and professional rules and principles for clinicians and related to the standard and the delivery of healthcare. The AMA [50] statement makes a number of demands of the Australian government in relation to the reform of detention policies. It covers issues such as access to care, who should deliver healthcare services and competency. It also gives specific advice on hunger strikes. This statement re-enforces calls for clinical autonomy made in the AMA Code of Ethics [45], stating that “[m] edical practitioners should … act in the best interests of the patient” and that “[d] octors should have the freedom to exercise their professional judgement in the care and treatment of their patients”. This statement provides scope for advocacy and also discusses the conduct expected in relation to confidentiality and privacy. Like the AMA Code of Ethics (2016), the AMA position statement (2015) reiterates many of the same standards, calling for doctors to “not allow lower standards of care to be provided” and that the standard of care provided should reflect that which would be applied in the broader Australian community. The APS [52] statement is framed far more generally. Beyond the calls for reform it makes, it outlines roles that psychologists could take up in supporting refugees and asylum seekers, including advocacy and research. It then makes seven recommendations for psychologists relating to clinical independence, professional competency, advocacy and cultural awareness. The APS [52] also encourage psychologists to engage in broader social and political action along with promoting the rights, health and wellbeing of asylum seekers and refugees.

### What clinician testimony says about healthcare in detention

Testimony from clinicians who formerly worked within immigration detention centres can be found throughout the literature, the media and in a number of inquiries. While it more often than not stands in stark contrast to the guidelines outlined about, there are some points of convergence. Like the codes and position statements discussed above themes of advocacy and clinical independence are prominent. Clinicians have also exposed the harms of detention and have long called for reform. Beyond this however, how clinicians go about the resolution of day to day dilemmas is starkly different to the principles, standards and conduct recommended in the above codes and position statements. The guidance found within codes, to place patients first, advocate where appropriate and guard clinical independence need to be seen against what has been described by many as a near futility in delivering care.

Dr. Peter Young, Psychiatrist and former Medical Director of International Health and Medical Services (IHMS) wrote and spoke extensively about his experiences in managing healthcare services across the detention network and his dealings with the immigration department. At the time he was the most senior figure who had worked in the system to condemn it. Here he discusses the impact of the Australian government’s policy of deterrence, how this was antithetical to health and healthcare and why treatment was largely ineffective:

… you can’t mitigate the harm, because the system is designed to create a negative mental state. It’s designed to produce suffering. If you suffer, then it’s punishment. If you suffer, you’re more likely to agree to go back to where you came from. By reducing the suffering you’re reducing the functioning of the system and the system doesn’t want you to do that … Everybody knows that the harm is being caused and the system carries on. Everybody accepts that this is the policy and the policy cannot change. And everybody accepts that the only thing you can do is work within the parameters of the policy [53].

A number of other clinicians have discussed how they delivered treatment and negotiated the day to day restrictions facing healthcare. Guy Coffey, a clinical psychologist and lawyer wrote about his experiences treating detained refugees and asylum seekers in the community, while working for Foundation House (formerly the Victorian Foundation for Survivors of Torture). While he discusses a range of issues, almost all appear to be underpinned by the tension he faced in navigating and mediating the restrictions placed on his role:

Treatment recommendations may fail to consider patients’ broader interests and may be confined by policy goals within the detention environment. In other words, treatment recommendations may be formulated for “what is possible” given the current circumstances rather than what is in a patient’s best interests. In many cases, the action needed to assist in mental health treatment and recovery is it quite obvious, with the best option for most patients being that they are removed from the detention environment. The tensions though, in how far one takes recommending alternative arrangements, are obvious. Not to do so is to remain silent about a significant and perhaps determinative effect on the detainee’s prognosis. Some might argue that it is to collude with the convenient lie that extended detention can be psychologically benign. Conversely, making recommendations about services that are not available, or regularly insisting on the need for the detainee to be released, risks detracting from the measures that can be taken immediately. It is an approach that runs the risk of having recommendations dismissed as advocacy, of alienating the IDC [immigration detention centre] management and the Department and therefore jeopardising the relationship between the IDC and the mental health service, and of leaving the IDC health staff feeling helpless [54].

Coffey’s [54] testimony also speaks to the precarious position of advocacy within immigration detention. Dr. Nick Martin, a general practitioner who was a senior medical officer on Nauru discussed similar concerns about advocacy and the issues this raised about putting the interests of his patients first:

Activism was stamped on incredibly quickly. It was seen as the greatest crime to be considered an advocate; it was to invite a swift cancellation of your visa and non-renewal of your contract. What was meant by ‘advocate’ was never explained. It seemed to me that our primary concern had to be the patient, and to push for the best appropriate treatment for them. If that was advocacy then surely it was what we did every day as doctors or nurses [36].

Others have concluded that the delivery of healthcare within immigration detention is simply futile. Almost 15 years ago, a healthcare professional provided a testimony at the People’s Inquiry into Immigration Detention [14] which included the following statement:

You could have the Rolls Royce of mental health services in Baxter and I don’t think it would make a scrap of difference, because the environment is so toxic that you can’t treat anything meaningfully. I think that half a dozen of the most damaged people that I’ve ever seen are the adults that I’ve seen in Baxter and Woomera, both parents and single men. The thing is that it is all caused by being in detention. Provided you get them in time, you take these people out of detention and they’re not depressed any more. Of course the interpretation of that from DIMA [Departent of Immigration and Multicultural Affairs, now the Department of Home Affairs] is to say they’re putting it on, “Isn’t it convenient for them, the thing that was going to cure them from their depression is taking them out of detention.” The reason it’s going to cure them is because detention is a place that drives people mad and yeah, they want to get out of the place that is driving them mad.

Similarly, Harold Bilboe, a psychologist who formerly worked at Woomera detention centre was quoted during the first National Inquiry into Children in Detention:

No matter how much I worked with the clients, I couldn’t change the cause of the behaviour, the course of their stress, it’s like having a patient coming into the hospital with a nail through the hand and you are giving them pethidine injections for pain but you don’t remove the nail. That’s exactly what is happening in Woomera. You’ve got people down there with nails through their hands, we’re holding them, we’re not treating the cause. So, the trauma, the torture, the infection is growing. We are not treating it, we’re just containing it. Eventually when those people return to their homelands, if they don’t get temporary visas, they are going to carry that with them [55].

### Reflections on the chasm between guidance and the delivery of healthcare

Some reflections are warranted on the obvious discrepancies between the guidance outlined above and the issues related to the delivery of healthcare as outlined by clinician testimony.

The testimony from clinicians supports my earlier assertion that Australian immigration detention alters the nature and scope of healthcare. Most fundamentally, clinicians working in detention support a system, both actively and passively, that is antithetical to the health and wellbeing of those who they are supposed to be helping. There are no solutions for this in the guidance outlined above and for those who have worked in the system, there is little that can be done to mitigate the harm promoted by these policies. How do we reconcile this position with calls from professional bodies to practice at the “highest level of excellence” [49] to “not allow lower standards of care to be provided” [50]?

Much of the guidance discussed above takes on new meaning when applied in an immigration detention settings. Guidance calls on clinicians to “[u] phold professional autonomy and clinical independence … without undue influence by individuals, governments or third parties” [45] and to “refrain from engaging in multiple relationships that may … impair their [psychologists] competence, effectiveness, objectivity, or ability to render a psychological service” [46]. Compare this to testimony from Coffey [23] which demonstrates how difficult this is in practice. In Australian immigration detention centres, placing the interests of the patients first in every instance may not even be desirable. Also noted by Coffey [23], what were perceived to be unreasonable requests could lead to repercussions from security contractors and the immigration department. Thus in some cases, on balance it could be appropriate to abide by the restrictions put forth by centre management. In other cases it could be more appropriate to advocate for those detained, or act subversively when it presents minimal risk. Closely related to this point is how advocacy was negotiated. All testimony indicates that in addition occupying an ambiguous place within detention, advocacy was frequently “dismissed” [54] while activism was “stamped on incredibly quickly” [36].

These well documented shortcomings, along with the well documented issues in the delivery of healthcare have gone largely unaddressed, with only the RACP [51] acknowledging the “tension in defining a standard of care” and the RANZCP (2016) raising concerns about psychiatrists’ ability to “provide high quality mental healthcare and to practice ethically”. More fundamental questions also remain; what are a patients best interests mean in this context? Should clinicians advocate for their patients release or simply pursue care as usual? The RANZCP [48] Code of Ethics states that “[p] sychiatrists have a duty to advocate for services and take appropriate action”. How this should be done and what “appropriate action” entails remain open to interpretation. Finally, how should clinicians guard the human rights and dignity of their patients, within a system where these are deliberately violated?

A final related point, that wasn’t discussed in clinical testimony above, is whether Australian immigration detention constitutes cruel and degrading treatment or even torture. There is a growing number of experts and academics that have raised concerns that these policies constitute cruel and degrading treatment [12], crimes against humanity [23] and even torture [24, 56, 57]. The AMA [45] and RANZCP [48] both explicitly call for doctors to refuse to “countenance, condone or participate” [45] in cruel and degrading treatment or procedures. In addition to failing to provide guidance for the day to day delivery of healthcare and leaving a number of fundamental questions unanswered, there should be ongoing discussion about whether clinicians should work in these environments at all and the possibility of a boycott.

Before discussing possible alternatives to the above codes and position statements, it is necessary to deal with some of the limitations and potential objections of my analysis. First, I have only discussed each code and position statement briefly, I have also lumped a number of professions together. Obviously each document has its relative strengths and weaknesses, each is intended for a different audience and profession. I have not attempted to focus my attention on one code or one profession for a number of reasons and this at least to some degree, comes at the expense of a more focused critique. This is to my knowledge the first article to critically reflect on the guidance that is available for clinicians working in Australian immigration detention. Furthermore, while future research can and should explore the relative strengths and weaknesses of each code or position statement, it is unnecessary to support my argument, namely that current codes and position statements fail to guide ethical decision making in Australian immigration detention. Second, my analysis cannot account for how this guidance is utilised at different times under different circumstances. Some principles may be easier to uphold and under certain circumstances, and at times, it may be possible to act consistently with the guidance contained in these instruments. I am therefore not suggesting that codes and position statements are completely redundant or that they fail to provide guidance in all circumstances. What I hope to have illustrated is that codes and guidelines fail to help in the overwhelming majority of cases and offer no means to address the well documented rights abuses that result from these policies. Finally, the above codes serve other purposes, beyond providing guidance. Codes can also be used as aspirational, educational or regulatory devices [58]. While it could be argued that the codes and position statements above do any of these things, with the exception of the RACP (2015) all explicitly set out to provide guidance. Regardless of this, and regardless of whether this is accepted, the disparity between guidance and the delivery of healthcare remains.

## Conclusions

### What are the alternatives?

While many practical questions remain unanswered, the disparity between codes of ethics and the delivery of healthcare in Australian immigration detention speaks to larger, more fundamental issue related to codes of ethics and the guidance they provide. When approached as a “set of principles or rules which are established by a professional body” [31] and under the assumption that if guided by these principles behaviour will be ethical, Dawson [59] argues codes run into two major problems. First, codes of ethics cannot account for previously unenvisioned situations. No code can account for all possible future scenarios, nor can any principle be applied in all potential circumstances. Practically this means that clinicians may be placed in a situation where “some ethical response is appropriate, but none of the rules seems to be relevant” [31]. Second, principles and statements found within codes can conflict. Both of these shortcomings together not only result “in problems when it comes to unenvisioned situations and dilemmas, but also in knowing when it is appropriate to apply a principle, and knowing which one is relevant in that situation” [31]. As an alternative to this ‘outside-in’ account of ethics, Dawson [31] goes on to suggest an alternative approach, namely a cognitivist account of ethical conduct:

… the hallmark of ethical action, is not the following of a certain rule, but having the flexibility to respond to the unique circumstances of a particular moment. Ethical action is not to be judged by how closely the agent mirrors an abstract set of rules, but by their ability to use the experience they have; to be open to new experience, advice, and criticism; and to be receptive to new ideas, and clients’ and colleagues’ attitudes and opinions … The idea of moral agency on this view becomes a radically dynamic one, an active seeking of the most appropriate action for those particular circumstances.

This approach looks beyond codes and position statements to a more dynamic, responsive form of ethical decision making. It allows far greater flexibility in responding to new situations or situations where ethical principles would otherwise conflict. Such an approach would allow clinicians to examine the unique elements of each situation and the trade-offs that come with it. How might a cognitivist approach be applied to facilitate ethical decision making Australian immigration detention centres? Below I will discuss some possible ways forward.

The first possibility is looking at present codes and how to improve these. There is certainly scope for this. We could look to close the chasm, acknowledging the shortcomings in the delivery of healthcare and clinicians compromised roles within the system. This of course does not mean that codes need to set lower standards, they could still contain aspirational standards, but specific attention is needed to the circumstances found within Australian immigration detention and how this fundamentally changes the delivery of healthcare. Also a possibility is that codes are reframed more broadly, only outlining overarching standards or principles. This would provide clinicians’ greater flexibility in responding when faced with new circumstances or in situations where more narrow principles would otherwise clash. Above, there were substantial differences in the framing of codes and position statements. For example, the International Council of Nurses Code of Ethics (2012) and the APS [52] position statement are arguably framed most broadly than others discussed here. While this may overcome some of the issues relating to unforeseen situations and conflicting ethical principles, when framed broadly codes are likely to provide little guidance.

A second possibility is that professional bodies refrain from providing any guidance at all and instead make demands of the government or outline what reform should look like. As discussed above, the RACP [51] is the only professional body to take this position, stating explicitly: “[t] his statement does not provide recommendations regarding detention health facilities, as the evidence shows held detention has a significant and detrimental impact on health and wellbeing, and the RACP does not condone held detention”. It could be argued that this approach is most consistent with Dawson’s [59] cognitivist account, allowing greater scope for clinicians to respond flexibly and “to the unique circumstances of a particular moment”. It could also be argued that refusing to provide advice would have broader implications, delegitimising these policies and making a statement that ethical conduct within these environments is simply not possible. If such an approach were taken however we may lose some of the other useful functions of codes, their simplicity and capacity to promote consistency across a profession [59] or their use as aspirational, educational or regulatory devices [58].

There is another possibility; looking beyond codes of ethics for guidance. Such an approach could address many of the shortcomings discussed above and could be used with existing (or amended) codes. One example could involve peer supervision and advice made available to those who work within immigration detention centres. This could be provided over the phone or online and thus provide both reactive and proactive support in relation to clinical and ethical decision making. Such an approach could assist clinicians in mediating conflicts and dealing with unforeseen circumstances, providing a more dynamic way resolve these dilemmas. Such an approach would have other benefits, it also would offer a degree of oversight and mediate some of the strong institutional forces that shape clinical and ethical decision making within detention centres.

Over two decades codes of ethics and position statements have contributed little to improving clinical practice within detention, there are however other ways, these should be seriously considered by professional healthcare bodies in Australia.

This leads to a final point, namely that even if a better approach to ethical and clinical guidance were adopted, any improvements in the health of those detained and in the delivery of healthcare would likely be marginal. Briskman and Zion (61) are correct in their assessment that, “a focus on maintaining and incrementally improving the system is vexed and the aspiration must be the abolition of the detention system”. While all professional bodies call for major reform, little is said about how such reform should be pursued. Beyond clinical and ethical guidance, there is scope to extend existing guidance to outline the role the healthcare community should play in social and political change. This appears to be the only way to truly resolve these dilemmas and protect the rights of refugees and asylum seekers in Australia.

## Data Availability

Not applicable

## Abbreviations

AMA: Australian Medical Association
APS: Australian Psychological Society
DIMA: Department of Immigration and Multicultural Affairs
IDC: Immigration Detention Centre
IHMS: International Health and Medical Service
RACP: Royal Australian College of Physicians
RANZCP: Royal Australian and New Zealand College of Psychiatrists
